# Active surveillance of carbapenemase-producing Enterobacterales using genomic sequencing for hospital-based infection control interventions

**DOI:** 10.1017/ice.2023.205

**Published:** 2024-02

**Authors:** Andie S. Lee, Leanne Dolan, Frances Jenkins, Bernadette Crawford, Sebastiaan J. van Hal

**Affiliations:** 1 Departments of Infectious Diseases and Microbiology, Royal Prince Alfred Hospital, Sydney, Australia; 2 Sydney Medical School, University of Sydney, Sydney, Australia; 3 Infection Control Unit, Royal Prince Alfred Hospital, Sydney, Australia; 4 Department of Microbiology, Royal Prince Alfred Hospital, Sydney, Australia

## Abstract

**Background:**

Whole-genome sequencing (WGS) is increasingly used to characterize hospital outbreaks of carbapenemase-producing Enterobacterales (CPE). However, access to WGS is variable and testing is often centralized, leading to delays in reporting of results.

**Objective:**

We describe the utility of a local sequencing service to promptly respond to facility needs over an 8-year period.

**Methods:**

The study was conducted at Royal Prince Alfred Hospital in Sydney, Australia. All CPE isolated from patient (screening and clinical) and environmental samples from 2015 onward underwent prospective WGS. Results were notified to the infection control unit in real time. When outbreaks were identified, WGS reports were also provided to senior clinicians and the hospital executive administration. Enhanced infection control interventions were refined based on the genomic data.

**Results:**

In total, 141 CPE isolates were detected from 123 patients and 5 environmental samples. We identified 9 outbreaks, 4 of which occurred in high-risk wards (intensive care unit and/or solid-organ transplant ward). The largest outbreak involved Enterobacterales containing an NDM gene. WGS detected unexpected links among patients, which led to further investigation of epidemiological data that uncovered the outpatient setting and contaminated equipment as reservoirs for ongoing transmission. Targeted interventions as part of outbreak management halted further transmission.

**Conclusions:**

WGS has transitioned from an emerging technology to an integral part of local CPE control strategies. Our results show the value of embedding this technology in routine surveillance, with timely reports generated in clinically relevant timeframes to inform and optimize local control measures for greatest impact.

Antimicrobial resistance is a major threat to patient safety. Carbapenemase-producing Enterobacterales (CPE) is considered one of the most important classes of multidrug-resistant organisms.^1–3^ These pathogens can cause large clonal hospital outbreaks,^4^ and they exhibit relative ease in transferring plasmid-based antibiotic-resistance gene elements across bacterial species. The increasing hospital burden of CPE acquisition from cross transmission may then lead to higher rates of invasive infections, with attributable mortality ranging between 29% and 75%.^5,6^

In Australia, other than imipenemase (IMP)-4, which has established low-level endemicity, CPE is relatively uncommon. “New” cases are often identified in screening samples from patients who have had healthcare contact in countries with a higher prevalence of CPE.^7^

Whole-genome sequencing (WGS)^8,9^ is currently considered the typing method of choice and is an important adjunct to informing CPE control efforts.^10^ In Australia, CPE sequencing has come under the remit of public health laboratories that traditionally use a centralized approach to testing.^10,11^ The greatest strength of using a public health framework is the public health infrastructure, particularly pertaining to data collection, which can in turn inform overarching policy at a national level.

Interventions aimed at containing CPE, however, remain predominantly at the hospital level, where timely outbreak identification is critical to minimize spread. In principle, a centralized WGS approach should be able to respond to local needs, through timely reports and feedback, but this is not always feasible due to competing priorities. These delays, in conjunction with the iterative approach required to classify outbreaks, highlight the potential deficiencies with a centralized WGS approach, namely its suboptimal responsiveness to local needs.

Royal Prince Alfred Hospital established a local CPE WGS surveillance program in 2015. Here, we describe the utility and strengths of a comprehensive, prospective, real-time, local sequencing service to promptly respond to facility needs over the first 8 years of the program.

## Methods

### Setting

Royal Prince Alfred Hospital is a 920-bed, quaternary-care, referral hospital in Sydney, Australia, with a large intensive care unit (ICU) as well as solid-organ (liver and kidney) transplantation, hematopoietic stem-cell transplantation, and pelvic exenteration services. The ICU had 54 beds with 12 single rooms distributed over 4 units until it was reconfigured during 2021 to have 50 beds with 30 single rooms.

### Active CPE surveillance

All patients in the ICU underwent active surveillance for CPE with rectal swabs collected on admission, weekly, and on discharge from ICU. Patients who had had healthcare contact in another country in the previous 12 months and all interhospital transfers within Australia were also screened for CPE on admission. Point-prevalence surveys were undertaken every 3 months for high risk (solid-organ transplant and hematology) wards and in the setting of an identified cluster (with or without environmental sampling). CPE isolation from a sample taken ≤48 hours was defined as community acquired, and CPE isolation from a sample taken >48 hours after hospital admission was defined as hospital acquired. Cases in which CPE was detected from a sample collected ≤48 hours after admission from patients who had represented within 90 days of their previous hospitalization without interaction with another healthcare facility were also classified as hospital acquired.

### Laboratory surveillance and screening for carbapenem non-susceptibility

The CPE screening samples were plated onto the chromogenic selective media Brilliance CRE Agar (Thermo Fisher Scientific, Waltham, MA) with colonies identified as Enterobacterales using matrix-assisted, laser desorption ionization, time-of-flight mass spectrometry (MALDI-TOF MS, Bruker Daltoniks, Billerica, MA). All screening and clinical isolates underwent susceptibility testing using the Vitek 2 (AST-N246 cards, bioMèrieux, Marcy-l’Étoile, France). Subsequent PCR testing was performed on isolates with a meropenem minimum inhibitory concentration (MIC) ≥0.5 mg/L, previously using the MT-PCT AusDiagnostics (Sydney, Australia) CRE (16-Well) kit targeting GES, IMI, SME, KPC, NDM, IMP, VIM, OXA-23, −48, −51, and −58–like groups. This panel was replaced in 2020 with the Xpert Carba-R test (detects KPC, NDM, VIM, OXA-48, and IMP) on the GeneXpert instrument (Cepheid, Sunnyvale, CA).

### Whole-genome sequencing (WGS)

From 2015, WGS was performed on all Enterobacterales isolates with an increased meropenem MIC within 5–7 days of identification. DNA from a single colony was extracted using EZ1 DSP Virus Kit on the EZ1 Advanced XL (Qiagen, Hilden, Germany). DNA libraries were generated using the Illumina DNA prep kit (Illumina, San Diego, CA), and sequencing was performed on the Illumina Miseq or iSeq100 platform according to the manufacturer’s instructions.

### Bioinformatic analysis

Basic local alignment search tool (BLAST)–based software (versions are not shown because these changed over the study period) was used to determine multilocus sequence type (MLST) with antimicrobial resistance genes determined using amrfinder^12^ on assembled contigs using spades^13^ following base quality trimming using fastp.^14^ When 2 or more sequences of the same species with the same CPE gene and sequence type were detected, a clustering analysis was performed. This consisted of mapping the trimmed reads to a reference using bwa mem with SNPs inferred by freebayes (using an allele frequency of 0.9) and consensus was called using bedtools following the masking of indels. Phylogenetic trees were generated with iqtree.^15^ Plasmid(s) were reconstructed from assemblies using MOB-suite^16^ followed by all versus all comparison using blastn.

### Outbreak determinations

As cases were identified, they were initially assessed by the infection control unit in the absence of WGS data. When 2 or more isolates of the same species harboring the same CPE gene were detected in patients with epidemiological linkages (ie, temporal or geographical overlap within the hospital), a possible outbreak was declared.

WGS data were generated and analyzed regularly in real time. Based on genomic data, a probable outbreak was declared if sequences of the same species from 2 or more patients had the following characteristics: (1) shared the same MLST type; (2) shared the same CPE resistance gene(s); (3) were located within the same cluster by ClusterPicker version 1.2.5 specifying >95% bootstrap support and a genetic distance of <1%; and (4) were within an arbitrary threshold of <100 SNPs using a species-specific mapping approach. This SNP threshold was chosen to maximize the sensitivity of outbreak detection and to circumvent possible analysis limitations, which included reference choice, filtering of non-core sites (ie, sites not present in all isolates), and need for routine masking of recombinant sites. Provided that the reconstructed plasmid harbored the same CPE gene, a possible plasmid transfer event was defined if 2 or more reconstructed plasmids had similar observed structures (ie, identity and coverage >95%) when aligned.

**Figure 1.**
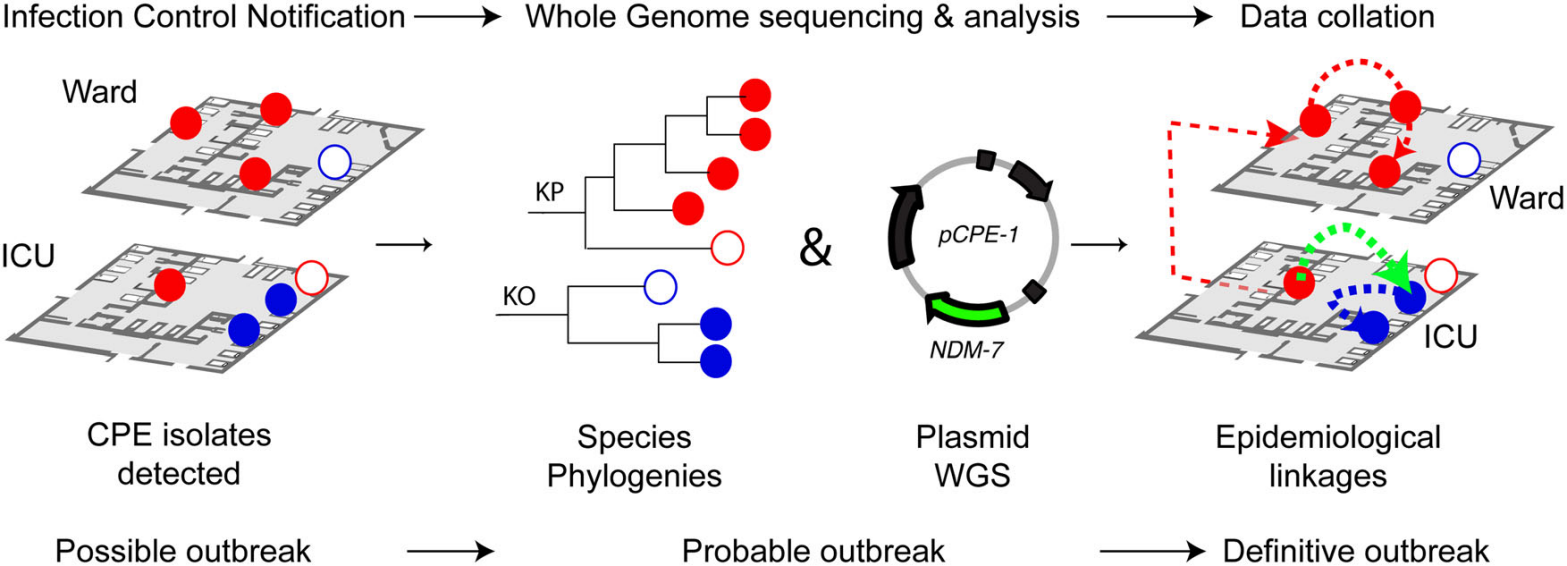
Carbapenemase-producing Enterobacterales (CPE) surveillance workflow (see text for details). CPE isolates are notified to the infection control unit by means of active and passive patient surveillance. If >1 isolate from patients with epidemiological linkages is detected, a possible outbreak is declared. All isolates are sequenced locally using Illumina technology and analyzed by species. A probable outbreak is established when genomically linked isolates or matching plasmids harboring the same CPE gene are detected (see text for details). An outbreak is confirmed when there are both genomic and epidemiological linkages. Open circles show unrelated isolates based on genomic criteria. Note. CPE, carbapenemase-producing Enterobacterales; ICU, intensive care unit; KO, *Klebsiella oxytoca*; KP, *Klebsiella pneumoniae*; NDM-7, *bla*_NDM7_; pCPE, plasmid harboring the CPE gene; WGS, whole-genome sequencing.

Outbreaks were confirmed when both genomic and epidemiological linkages were identified based on the criteria above. In instances where only genomic linkages were established, further detailed review of patient bed movement, geographical relationships with previous cases (to exclude potential outbreaks from environmental reservoirs) and staff interactions was undertaken prior to excluding an outbreak (Fig. 1).

### Infection control interventions

On notification of a CPE case, there was a staged infection control response (Table 1). First stage interventions included notification of the new CPE case to clinicians and implementation of IPC measures including institution of contact precautions, patient isolation, environmental cleaning, and contact tracing. Once identified as CPE colonized or infected, patients were presumed to be colonized for all future admissions and infection control precautions were continued. Where transmission was identified through epidemiological and genomic review, second-stage interventions were employed, including extending the scope of patient screening, assessing the need for environmental sampling, and convening an outbreak management team (OMT). Process audits tailored to review deficiencies detected during outbreak investigations included audits of hand hygiene compliance, cleaning of shared patient equipment, cleaners’ processes, adequate storage of medical equipment, and equipment investigations.

**Table 1.** Staged Infection Control Response to a Newly Identified Case of Carbapenemase-Producing Enterobacterales (CPE)

First stage interventions—Isolation, cleaning, and contact tracing
• Notification of new CPE case to relevant clinical area • Implementation of immediate infection prevention and control (IPC) measures: ○ Transmission-based precautions (contact precautions with use of gown and gloves) for patient care ○ Patient isolation in single room with en suite ○ Transfer of patient to appropriate clinical area where necessary ○ Enhanced cleaning with sodium hypochlorite (1,000 ppm) for terminal bedspace and shared patient equipment disinfection, and where able (ie, single room, open clinical area decanted of patients, or dirty utility room), hydrogen peroxide vapor decontamination to further reduce bioburden.^17^ • Additional notification to the hospital executive and patient flow team • Contact trace ○ Review of bed allocations and any procedures ○ Contact patients identified ▪ A contact defined as a patient located in an adjacent intensive care unit bed or who shared a bed bay or bathroom with confirmed CPE case for 24 hours or more^18^ ▪ CPE screening on day 0, 4 and 7 after last exposure to case ▪ If case identified ≥7 days after exposure, 2 CPE screens at least 24 hours apart

Second stage interventions—Extended screening, environmental sampling and convening the outbreak management team
• Ongoing IPC investigation based on correlation of microbiological results and genomic analysis of current and historic cases, to determine whether transmission suspected or confirmed • Where transmission identified, screening extended to wider scope of contacts including point-prevalence screening of all patients in unit/ward within specific period • Where transmission confirmed, outbreak management team established ○ To facilitate communication and management between stakeholders, including importance of antimicrobial stewardship ○ Including executive managers, nurse unit manager, attending medical consultant, infection prevention and control nurses, and infectious diseases physicians ○ In compliance with state guidelines^18^ • Clinical areas reviewed to determine need for environmental and equipment sampling • Routine monitoring of compliance with interventions such as enhanced cleaning of environment and associated equipment • As required, audits of local unit processes to ensure no lapses in IPC practices

No formal ethics review was necessary because screening, data collection, and genomic analysis were introduced as part of routine infection prevention and control surveillance activities and patient-identifiable data were not reported.

## Results

### Characteristics of CPE isolates

**Figure 2.**
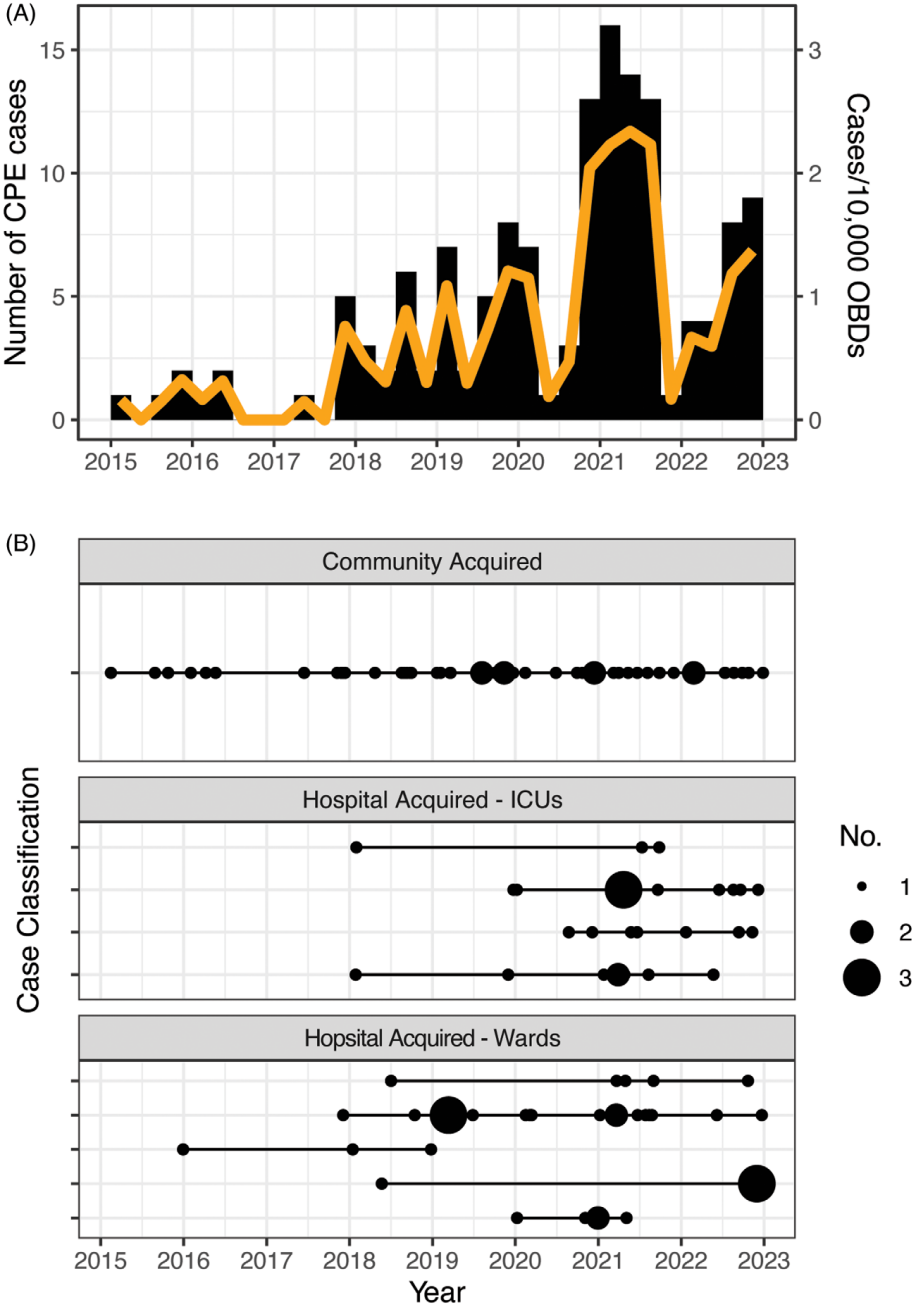
(A) Histogram plot of carbapenemase-producing Enterobacterales (CPE) cases per quarter from 2015 to 2022 with rate of CPE per 10,000 occupied bed days (OBDs) on the secondary y-axis depicted by the orange line. (B) Case classification or place of CPE acquisition with locations depicted only if they had at least 2 events across the period. Community-acquired cases include those patients transferred from another healthcare facility after their CPE acquisition. The number of cases per quarter are indicated by the size of the circle.

Between January 1, 2015, and December 31, 2022, 174 Enterobacterales isolates with an increased meropenem MIC were detected, with 164 isolates (94%) available for sequencing. After WGS, 23 isolates were excluded because no known CPE gene was detected. The remaining 141 isolates were from 5 environmental and 136 patient samples. Among the 136 patient samples, obtained from 123 unique patients, there were 44 clinical and 92 screening samples. Following a substantial increase in the overall number of CPE cases over time, a marked reduction in cases occurred toward the end of 2021 and the first quarter of 2022, followed by increasing CPE numbers in late 2022 (Fig. 2A).

In total, 51 patients (41%) acquired CPE in the community or other healthcare facility and 72 patients (59%) acquired CPE in our facility. The ICU and the solid-organ transplant unit accounted for most hospital (53 (74%) of 72) CPE acquisitions (Fig. 2B). The most common CPE genes encountered were *bla*_NDM_ (38%) followed by *bla*_IMP_ (33%) and *bla*_OXA_ (15%). Also, 11 isolates contained >1 CPE gene; combinations included NDM-IMP, NDM-KPC, NDM-OXA, and IMP-OXA. Of the 12 Enterobacterales encountered, 3 species (*Klebsiella pneumoniae, Escherichia coli*, and *Enterobacter cloacae*) accounted for 70% of isolates (Supplementary Table 1 online).

### WGS testing characteristics

Turnaround time for WGS results (from sample collection to genomic data interpretation) decreased over the 8-year period from a median of 14 days (interquartile range [IQR], 10–21) to a median of 9 days (IQR, 6–12). This improvement occurred after the optimization of workflows between sections of the laboratory and the infection control unit and the acquisition of an Illumina iSeq100 instrument, which allowed for smaller run sizes.

### Description of outbreaks

Over the 8-year period, 9 outbreaks were confirmed by WGS (involving 2–16 patients), 4 of which involved high-risk wards (ICU and/or the solid-organ transplant ward). The outbreaks followed 1 of 2 predominant patterns.

The first pattern (n = 7 outbreaks) was characterized by intermittent sporadic isolation of genomically linked CPE isolates from hospitalized patients without overlap in time in their hospital stays. Epidemiological linkages were based on patients having shared the same clinical area or ward, sometimes weeks to months apart, suggestive of an environmental reservoir. These events were exclusively observed with our endemic CPE, *bla*_IMP-4_, and would have been defined as new isolated introduction events in the absence of longitudinal sequencing data. For example, for one such outbreak, 2 isolates detected 6 months apart were genomically linked. Extensive sampling of surfaces and equipment (n = 116 samples) over a 1–2-week period detected CPE from 4 samples: high-touch areas in patient rooms (n = 1), shared patient equipment (patient hygiene aide, n = 1), and sink drain cultures (n = 2) from clinical hand basins and dirty utility rooms.

**Figure 3.**
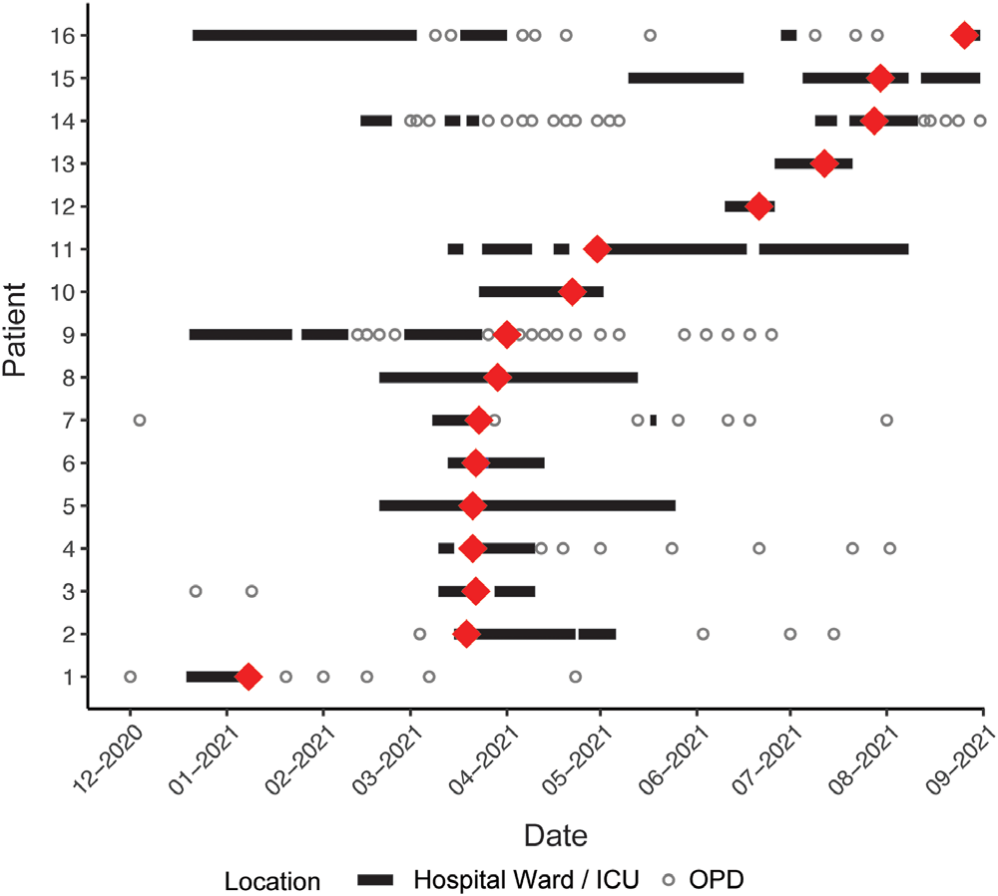
NDM-7 outbreak. Patient hospital admissions and outpatient visits are indicated by the black line and open grey circles, respectively. Red diamonds reflect dates of first carbapenemase-producing Enterobacterales detection. All patients were seen in the same OPD clinic with overlap of patients in the same consulting rooms. Note. ICU, intensive care unit; OPD, outpatient department.

The second outbreak pattern (n = 2 outbreaks) involved admission of a single CPE-colonized patient (the index case) followed by extensive onward transmission. One such outbreak with NDM-7 involved 16 patients (including 4 clinical and 16 surveillance isolates) over 250 days (Fig. 3). The outbreak occurred across several locations in the hospital with cross transmission in outpatient settings, where there were points of possible interaction between multiple cases prior to detection of the outbreak, acting as a potential source of CPE reintroduction into the hospital. The implicated outpatient setting is a busy clinic with a multibed treatment room where invasive procedures (eg, renal biopsies) using shared patient equipment are performed, increasing potential transmission risk. Environmental sampling also confirmed the presence of an NDM-7–carrying *E. cloacae* from a dialysis port in the ICU. This led to a comprehensive review and introduction of enhanced infection control measures in the inpatient and outpatient settings (including PPE recommendations, dedicated bathroom facilities, and enhanced and targeted cleaning to include equipment or devices which had previously been overlooked). This outbreak was characterized by initial species clonality followed by interspecies plasmid exchanges and genomic drift over time.

## Discussion

Identifying epidemiological and genomic linkages between patient cases and environmental samples has been fundamental to understanding the source and extent of CPE outbreaks in our institution. The availability of timely data, providing evidence for and illustrating the nature of local transmission, has facilitated directed ongoing infection prevention and control response rather than broad, large-scale strategies that are costly and resource-intensive. Outbreak investigations have assisted in identifying areas for extended audits of cleaning and equipment function, which have in turn demonstrated a patient safety need for capital expenditure on equipment upgrade. Similarly, environmental samples that were genomically linked to patient outbreak isolates highlighted deficiencies in routine environmental decontamination protocols and supported refining cleaning and disinfection procedures.

The prospective, routine use of WGS for all newly identified CPE cases provided a comprehensive assessment of the transmission dynamics of this multidrug-resistant organism within our facility. The largest outbreak detected during the 8-year period involved Enterobacterales harboring an NDM gene, with interspecies transfer of the resistance plasmid. This finding is consistent with previous reports of this carbapenemase’s outbreak potential related to plasmid transfer.^19^ The addition of plasmid linkage analysis provided enhanced detection of the extent of clusters, identifying genomic links that would have been missed by relying on species-specific surveillance of CPE transmission. In addition, unexpected genomic links between patients led to further investigation of epidemiological links, uncovering the outpatient setting, where patients had repeated visits over time, as a common point of overlap. These findings prompted targeted infection control interventions in this setting.

An important aspect of the WGS service in our institution has been the responsiveness of the service to meet local needs. In addition to the onsite service facilitating timely results, local access to WGS has also had the advantage of ensuring that sequencing results are interpreted in the appropriate clinical context taking into consideration timelines (and rates of evolution), epidemiological data, and the knowledge of endemic or widespread epidemic transmission of CPE strains in the hospital or community. Consideration of information regarding epidemiological links is particularly important as WGS cannot determine the directionality of transmission events in identical isolates.^20^ The timeframe and contact information are crucial in determining transmission chains and the degree of certainty of transmission events.^21^ Conversely, genomic data also enabled us to exclude suspected cross-transmission events in some instances in which epidemiological links were found. These final determinations of the presence of an outbreak require a continuous iterative approach through close liaison and feedback between local clinicians and the WGS service.

The availability of genomic reports in real time was also instrumental in obtaining prompt executive support for infection control interventions. When hospital executives were presented with epidemiological and genomic evidence clearly demonstrating hospital acquired CPE transmission in real-time during a CPE outbreak, these reports were a catalyst in gaining their confidence and support for capital improvements to mitigate ongoing patient impact and costs associated with transmission. The strong genomic evidence has engaged the executive administration to consider and subsequently approve infection control recommendations for local improvements that require additional resources. These improvements have included support for new equipment for reprocessing of reusable equipment or transition to single-patient-use devices that are usually shared between patients and have been identified as a transmission risk.

Pathogen WGS is increasingly accessible to diagnostic laboratories with well-established, routine sequencing workflows.^22^ In addition to outbreak investigation, having this technology on site has allowed us to establish expertise and workflows in other applications such as antimicrobial resistance detection and metagenomics, which have increasing clinical utility. Running costs are similar whether WGS is performed centrally or locally. The largest hurdle remains interpretation of genomic data. One solution would be investing in a dedicated bioinformatician, which would result in increased per sample cost. Our CPE prevalence would not currently justify such an approach. We have overcome this hurdle by performing data analyses using freeware software tools with reporting performed by a credentialed microbiologist. An alternative approach would be to access an off-site WGS service with bioinformatic expertise, if timely results and close liaison with the local clinicians could be provided.

Unfortunately, prospective studies demonstrating and quantifying the beneficial impact of routine WGS implementation are limited.^23,24^ Most data are derived from retrospective studies, many of which reconstruct chains of transmission during outbreak investigations.^25,26^ However, data describing the prospective use of WGS to enable focused infection control measures are emerging for a number of multidrug-resistant organisms.^27,28^ Our study adds to the existing literature with a more in-depth focus on the control of CPE, and it is unique in that data were collected over a prolonged period. It remains difficult to prove whether WGS as part of routine surveillance results in a reduction in the number of healthcare infections. However, the increased granularity provided by WGS has allowed us to nuance our infection control strategies and potentially reduce overall intervention costs. Further data are required to determine the overall net saving, and this could be an area for future research. Studies demonstrating the cost-effectiveness of WGS for infection control and outbreak detection and management are starting to emerge^29,30^ and will help support implementation decisions.

In conclusion, over the past 8 years of our local experience with the use of routine, real-time WGS for CPE surveillance to inform outbreak management, it has become increasingly evident that WGS has transitioned from an emerging technology to an integral part of our local CPE control strategy. WGS is now an established tool for confirming or refuting outbreaks and providing a detailed understanding of the transmission of healthcare-associated pathogens. Our findings highlight the importance of access to timely results generated in a clinically relevant timeframe that can, in close collaboration between clinical and laboratory staff, help inform and optimize local control measures for greatest impact.

## Supplementary material

For supplementary material accompanying this paper visit https://doi.org/10.1017/ice.2023.205. click here to view supplementary material
