# Supplementary material for: Active surveillance of carbapenemase-producing Enterobacterales using genomic sequencing for hospital-based infection control interventions
Source: Infect Control Hosp Epidemiol. 2023 Sep 13;45(2):137–43. doi: 10.1017/ice.2023.205 (PMC10877539; doi:10.1017/ice.2023.205)
Supplement: Supplementary file 1 [file S0899823X23002052sup001.docx]

**Supplementary Material for manuscript**

**“Active Surveillance of carbapenemase-producing *Enterobacterales* using Genomic Sequencing for Hospital-Based Infection Control Interventions”**

**Supplementary Table 1. Clinical carbapenemase-producing *Enterobacterales* isolates identified at Royal Prince Alfred Hospital between 2015-2022**

| **Species** | **Gene** | **MLST^a^** | **n** |
| --- | --- | --- | --- |
| ***Citrobacter amalonaticus*** | NDM-7 & IMP-4 | Unassigned | 1 |
| ***Citrobacter freundii*** | IMP-4 | Novel | 1 |
|  | IMP-4 | ST22 | 4 |
|  | IMP-4 | ST8 | 1 |
|  | IMP-4 | ST95 | 1 |
|  | IMP-4 & OXA-48 | ST259 | 3 |
|  | NDM-7 | ST22 | 1 |
|  | OXA-48 | ST708 | 2 |
| ***Citrobacter koseri*** | IMP-4 | Unassigned | 1 |
| ***Enterobacter cloacae*** | IMP-4 | ST105 | 1 |
|  | IMP-4 | ST108 | 4 |
|  | IMP-4 | ST109 | 3 |
|  | IMP-4 | ST114 | 2 |
|  | IMP-4 | ST175 | 1 |
|  | IMP-4 | ST45 | 2 |
|  | IMP-4 | ST66 | 2 |
|  | IMP-4 | ST742 | 1 |
|  | IMP-4 | ST88 | 2 |
|  | IMP-4 | Unassigned | 1 |
|  | IMP-4 & OXA-48 | ST78 | 2 |
|  | NDM-7 | ST63 | 1 |
|  | NDM-7 | ST78 | 2 |
| ***Enterobacter hormaechei*** | IMP-4 | ST110 | 3 |
|  | IMP-4 | ST113 | 1 |
|  | IMP-4 | ST45 | 2 |
|  | IMP-4 | ST62 | 1 |
| ***Escherichia coli*** | IMP-26 | ST354 | 1 |
|  | IMP-4 | ST3580 | 1 |
|  | IMP-4 | ST5869 | 1 |
|  | IMP-4 | ST681 | 1 |
|  | NDM-1 | ST1193 | 1 |
|  | NDM-1 | ST83 | 1 |
|  | NDM-18 | ST4450 | 1 |
|  | NDM-5 | ST1284 | 1 |
|  | NDM-5 | ST167 | 3 |
|  | NDM-5 | ST196 | 1 |
|  | NDM-5 | ST405 | 2 |
|  | NDM-5 | ST410 | 1 |
|  | NDM-5 | ST5229 | 2 |
|  | NDM-5 | ST617 | 1 |
|  | NDM-5 | ST648 | 1 |
|  | OXA-181 | ST410 | 1 |
|  | OXA-181 | ST8489 | 1 |
|  | OXA-244 | ST3268 | 1 |
|  | OXA-48 | ST38 | 1 |
|  | OXA-48 | ST405 | 1 |
|  | OXA-48 | ST648 | 1 |
| ***Klebsiella oxytoca*** | IMP-4 | ST50 | 1 |
|  | NDM-1 | ST180 | 1 |
|  | NDM-1 | ST2 | 1 |
|  | NDM-1 | Unassigned | 2 |
|  | NDM-7 | ST2 | 3 |
|  | NDM-7 | Unassigned | 1 |
|  | NDM-7 & IMP-4 | ST350 | 3 |
|  | OXA-48 | ST2 | 1 |
|  | OXA-48 | ST257 | 1 |
| ***Klebsiella pneumoniae*** | IMP-4 | Novel | 1 |
|  | IMP-4 | ST1626 | 5 |
|  | IMP-4 | ST2118 | 1 |
|  | IMP-4 | Novel | 1 |
|  | KPC-2 | ST258 | 1 |
|  | KPC-3 | ST307 | 2 |
|  | NDM-1 | ST11 | 1 |
|  | NDM-1 | ST1418 | 1 |
|  | NDM-1 | ST219 | 5 |
|  | NDM-1 | ST394 | 1 |
|  | NDM-1 | Unassigned | 1 |
|  | NDM-4 | ST15 | 1 |
|  | NDM-4 | ST16 | 1 |
|  | NDM-5 | ST147 | 1 |
|  | NDM-5 | ST16 | 1 |
|  | NDM-7 | ST17 | 14 |
|  | NDM-1 & KPC-2 | ST11 | 1 |
|  | NDM-4 & OXA-181 | ST16 | 1 |
|  | OXA-181 | ST25 | 1 |
|  | OXA-181 | ST336 | 1 |
|  | OXA-232 | ST16 | 1 |
|  | OXA-232 | ST2096 | 1 |
|  | OXA-232 | ST661 | 1 |
|  | OXA-48 | ST16 | 1 |
|  | OXA-48 | ST395 | 1 |
|  | OXA-48 | Unassigned | 2 |
| ***Klebsiella variicola*** | IMP-4 | ST616 | 1 |
| ***Morganella morganii*** | IMP-4 | Unassigned | 1 |
|  | NDM-1 | Unassigned | 1 |
| ***Providencia rettgeri*** | OXA-48 | Unassigned | 2 |
| ***Salmonella enterditis*** | NDM-1 | ST11 | 1 |
| ***Serratia marcescens*** | SME | ST78 | 1 |

^a^Unassigned: isolates with incomplete alleles across multi-locus sequence genes.
